# Factors influencing community participation in control and related operational research for urogenital schistosomiasis and soil-transmitted helminths in rural villages of Kwale County, coastal Kenya

**DOI:** 10.11604/pamj.2016.24.136.7878

**Published:** 2016-06-10

**Authors:** Jacinta Wairimu Macharia, Zipporah W Ng'ang'a, Sammy Michugu Njenga

**Affiliations:** 1 Institute of Tropical Medicine and Infectious Diseases, Jomo Kenyatta University of Agriculture and Technology; 2 Department of Applied sciences, Department of Academics, Deputy Vice Chancellor office, Southern and Eastern Kenya University, Kitui, Kenya; 3 Eastern and Southern Africa Centre of International Parasite Control Centre, Kenya Medical Research Institute, Nairobi, Kenya

**Keywords:** Urogenital schistosomiasis, helminthes, Kenya

## Abstract

**Introduction:**

helminthic infections caused by soil-transmitted helminths (STH) and schistosomes are among the most prevalent afflictions of humans who live in areas of poverty. An operational research was undertaken in 5 villages of Kwale County during a pilot control programme which included both the adults and school going children. Willingness of community members to participate in the treatment as well as in the research is critical. A cross sectional study sought to determine factors influencing community participation in control and related operational research and assess the treatment coverage for urogenital schistosomiasis and hookworms in rural villages of Kwale County.

**Methods:**

cross-sectional survey utilized quantitative and qualitative methods of data collection. A total of 220 households were recruited and household heads interviewed. Bivariate analysis was used to test association between different independent and dependent factors. Multivariate analysis was done using binary logistic regression to control for confounders and effect modification. Qualitative data was transcribed, coded and analyzed thematically.

**Results:**

religion and levels of income were significantly (P =0.04 and P = 0.026 respectively) associated with participation in the research and control programme, history of ever suffering from schistosomiasis and intestinal worms was found to be significantly (P = 0.008) associated with participation in the research. The study established that 82% (178) of the respondents received treatment for urogenital schistosomiasis and hookworms and 67% (146) of the respondents had participated in the research.

**Conclusion:**

this information will be useful in promoting health, enhancing learning and behaviour changes which will lead to increased community participation in similar disease control.

## Introduction

Human helminthic infections exhibit over dispersed distribution so that most individuals harbor just a few worms, with a few hosts harboring large worm burdens. As a rule, about 20% of the host population harbors approximately 80 percent of the worm population [1]. This over dispersion has many consequences, because heavily infected individuals are simultaneously at highest risk of disease and the major source of environmental contamination, mainly the intensity of infection that determines the severity of morbidity [2]. Transmission of schistosomiasis occurs when a human′s skin comes in contact with fresh water contaminated with schistosome-carrying snails [3] and schistosome parasites penetrate the skin. STH infections are transmitted through indiscriminate disposal of human and animal faeces, poor personal hygiene, and inadequate water supply. Recent studies conducted in Kwale County in coastal Kenya revealed high prevalence (41.7%) of hookworms (*Ancylostoma duodenale* and *Necator americanus*) and (18.2%) of urogenital schistosomiasis (S. haematobium) infections among adults which called for the need to design strategies for including adults in control programmes [4]. Success of prevention and control programmes of any disease becomes more effective through community participation and involvement with the target population [5]. Local knowledge, practices, culturally and socially patterned behaviors of the target population if well understood provides important insights in decisions making which are more sustainable and appropriate during programme implementation. Community health-promotion research previously had focused on the outcomes of capacity building interventions and not on barriers of interventions. This lack of attention results in poor understanding of community participation and other factors may affect program implementation and their relationship to program outcomes [6]. Clarifying these factors may help practitioners implement more effective community based interventions.

The present study sought to identify factors influencing community participation in control and related operational research for urogenital schistosomiasis and intestinal worms among communities living in Kwale County, Coastal Kenya. Community engagement and participation has played a significant responsibility in success of communicable disease control and elimination campaigns such as the elimination of schistosomiasis in Guangxi Province, China [7] which endowed in mass literacy classes with the aim of providing an extensive and long term benefits for health of communities. There is still lack of good understanding of programme staff working with communities in which programmes are being implemented which can lead to disagreements [8]. However, knowledge transfer through mutual sharing of experiences between communities and program staff has proven to be an effective mechanism in facilitating community participation [9] in these control programmes. Communities which are more vulnerable to communicable diseases due to either biological or non-biological factors needs empowerment to participate in disease control programmes in order to mitigate the risk and reduce their vulnerability to disease [10]. Previous investigations studied on local factors such as exposure (water contact behaviour), contamination (urination and defecation), attitudes and knowledge bearing on transmission and illness behaviour, which draws the need to address broader issues including socioeconomic, and health utilization. A study conducted in Zimbambwe revealed that community participation is a complex process upon which a multiplicity of social and cultural determinants has an impact [11]. If community participation becomes successful in disease control programmes it ought to be viewed as a mutual learning process where problems are identified and discussed and solutions shared among community members and staff.

## Methods

**Study area and study design:** the study was carried out in two villages (Mirihini and Mlafyeni) in Mwaluphamba Location, Matuga District, Kwale County [10]. A cross-sectional survey found the prevalence of urogenital schistosomiasis and hookworm infection among adult population in these rural villages to be 18.2% and 41.7%, respectively [10]. Kwale County is located south of Mombasa Island and borders Tanzania to the southwest and the Indian Ocean to the east. Kwale County covers an area of 1,043 square kilometers. The population of Kwale County based on 2009 census was estimated as to be 649,931 and 151,978 persons [11]. The study was a cross-sectional descriptive survey involving quantitative (through questionnaire) and qualitative research methods (through Focus Group Discussions and Key informant interview). The study population comprised of household heads from selected houses in the two villages selected (Mirihini and Mlafyeni) from the five villages in which an ongoing study in the area had selected (Kajiweni, Mlafyeni, Mirihini, Miatsani and Maponda). Approval to carry out the study was sought from KEMRI Scientific Review Committee (SSC) and Ethical Review Committee (ERC) as well as from the Board of Postgraduate Studies (BPS) in Jomo Kenyatta University of Agriculture and Technology. Only those participants who consented participated in the study.

**Sample methodology and study population:** the study population comprised of a sample size of 220 household heads from selected from the two villages. The quantitative sample size was determined as described by (Fisher et al., 1998) taking the prevalence to be estimated at 50%, with 95% level of confidence and 7% bound on the error of estimation. A non-response rate of 10% was allowed. The two villages were selected from the list of the five villages through simple random sampling. An updated list of the household heads from the selected villages was used to select 110 household heads from each village through systematic random sampling.

**Data collection tools:** structured questions with closed and open ended questions were administered to household heads in the selected households in the two villages by trained research assistants. Key informant interviews were also conducted to explore in-depth contextual data among specific personalities. Focus group discussions (FGDs) were conducted to obtain in-depth information on the subject matter.

**Data management and analysis:** data captured in questionnaires were coded and entered in Ms Access and analysis done using Statistical Package for Social Sciences (SPSS) 16.0. All variables were subjected to descriptive data analysis. Bivariate analysis was carried out to determine the relationship between community participation and associated factors using Pearson's chi-square test. P value less than 0.05 were considered significant. Focus Group Discussions were sorted manually according to themes and then discussed.

## Results

### Characteristics of the study population

Table 1 presents a description of the sample population as well as showing associations between demographic and socioeconomic factors with community participation. A total of 217 participants took part in the study 110 from Mirihini and 107 from Mlafyeni villages respectively. Close to half of the respondents did not have formal education 102 (47.5%), the study found that there was 100% participation of those who had obtained tertiary education despite being few in the community. Most of the participants were farmers 153 (70.5%) while 41 (18.9%) were unemployed (Table 1). A small proportion 10 (4.6%) had formal employment and 11 (5%) were self-employed. There was a significant relationship between the average monthly income earned by the respondents and their participation in research (χ^2^= 11.013, df =4, P = 0.026) with those earning higher income being likely to participate in the research compared to those who earned less. Multivariate analysis was carried out using binary logistic regression to control for confounders and effect modification. Variables with P < 0.05 in logistic regression were considered to have a significant association with participation in research. Enter method a procedure of variable selection in which variables in a block are entered in a single step was used to establish true predictors. The predictors of research participation were estimated by calculation of odds ratio (OR) and 95% confidence intervals and p < 0.05 was considered significant. Religion was significantly associated with participation in research for schistosomiasis and STH (P = 0.041), with Muslims being 0.485 (95% CI= 0.236-0.997) times more likely to participate in the research compared to Christians. Income was also significantly associated with the respondents participation in the research (P= 0.004). Average monthly income of 11USD to 57 USD also revealed an association (P= 0.068), with a majority of those who had earned average monthly income of 57 USD-114 USD being 5.7 [95% CI= 1.467-2.23] times more likely to participate in the research compared to those who earned above 114 USD and below 57 USD.

**Table 1:** Demographic and socio-economic characteristics of the surveyed households and relationship with participation in urogenital schistosomiasis (*S.haematobium*) and hookworm's research and control programme

Variables	Measurement	Total N = 217 n%	Participation in research n%	Non-participation in research n%	P value
**Gender**	Male Female	60 (27.6) 157(72.4)	39 (65) 107(68.2)	21(35) 50(31.8)	0.658
**Age**	< 20	5(2.3)	4(2.7)	1(1.4)	0.949
	20-30	69 (31.8)	47(32.2)	22(30.9)	
	30-40	57(26.3)	38(26)	19(26.8)	
	40-50	36(16.6)	25(17.1)	11(15.5)	
	50 and above	50(23)	32(21.9)	18(25.4)	
**Marital status**	Married	194(89.4)	128(87.7)	66(92.9)	0.362
	Separated	4(18.4)	3(2.1)	1(1.4)	
	Single	13(5.9)	9(6.2)	4(5.6)	
	Widowed	6(2.8)	6(4.1)	0(0)	
**Family size**	2 -5	27(12.4)	18(12.3)	9(12.7)	0.364
	5- 8	93(42.9)	67(45.9)	26(36.6)	
	8 – 10	45(20.7)	31(21.2)	14(19.7)	
	10 and above	52(23.9)	30(20.5)	22(30.9)	
**Education**	Non formal education	102(47)	62(42.4)	40(56.3)	0.331
	Primary complete	45(20.7)	35(23.9)	10(14.1)	
	Primary incomplete	53(24.4)	37(25.3)	16(22.5)	
	Secondary	15(6.9)	10(6.8)	5(7)	
	Tertially	2(1)	2(1.4)	0(0)	
**Religion**	Christian	38(17.5)	20(13.6)	18(25.4)	0.041
	Muslim	179(82.4)	126(86.3)	53(74.6)	
	Traditional religion	0(0)	0(0)	0(0)	
**Occupation**	Self-employed	11(5.1)	7(4.8)	4(5.6)	0.740
	Formal employment	10(4.6)	8(5.5)	2(2.8)	
	Un-employed	41(18.9)	25(17.1)	16(22.5)	
	Farmer	153(70.5)	105(71.9)	48(67.6)	
	others	2(0.92)	1(0.7)	1(1.4)	
**Income**	Below 1000	11(5.1)	2(1.37)	9(12.7)	0.026
	1001-5000	195(89.9)	135(92.4)	60(84.5)	
	5001-10000	5(2.3)	3(2.05)	2(2.8)	
	10001-20000	5(2.3)	5(3.42)	0(0)	
	Above 20000	1(0.46)	1(0.68)	0(0)	

**Figure 1:**
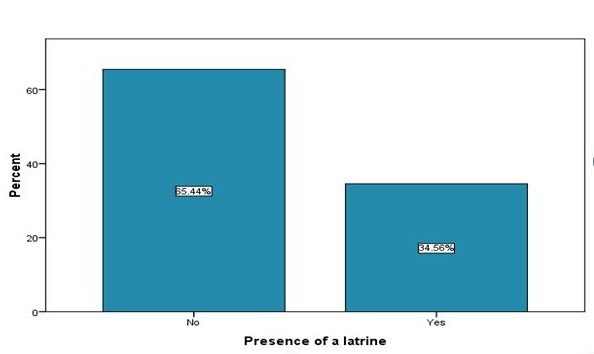
Distribution of respondents from the selected households by presence of latrines

Health seeking behaviour of participants on urogenital schistosomiasis and hookworms infections Among 217 respondents 57.1% (124) had a history of suffering from urogenital schistosomiasis (*S.haematobium*) and hookworm (*Necatus americanus* and *Ancylostoma duodenale*) infections. Children were the most vulnerable to these infections as stated by 213 (92.7%) of the respondents. An investigation to gather information whether the adults sought for treatment of urogenital schistosomiasis and hookworms revealed that 42.9% (93) of the respondents had never sought treatment while 57.1% (124) had sought treatment. There was a significant relationship between participation of the respondents in the research and having ever sought for treatment of schistosomiasis and hookworms (χ^2^= 5.628, df = 1 p < 0.05) with 73.3% (65) of those who had ever sought treatment participating in the research. A further investigation on why the respondents would not visit a health facility in the face of urogenital schistosomiasis and hookworms revealed that 193 (88.9%) was due to health facilities being too far away from home and no means of transport, 13(6.9%) it was expensive to seek treatment from a health facility and 9 (4.2%) indicated (they didn't have a reason, some did not trust the health workers and others feared to find out that they had the disease. Most of the households 142(65.4%) did not have a latrine (Figure 1). Although there was no significant relationship between having a latrine and having ever suffered from urogenital chistosomiasis and hookworms infection, a higher proportion of respondents who did not possess a latrine indicated they had suffered from hookworms and urogenital schistosomiasis than those who had latrine (χ^2^=0.287, p > 0.05).

### Participation in the treatment and research of urogenital schistosomiasis and hookworm infections by the participants

**Figure 2:**
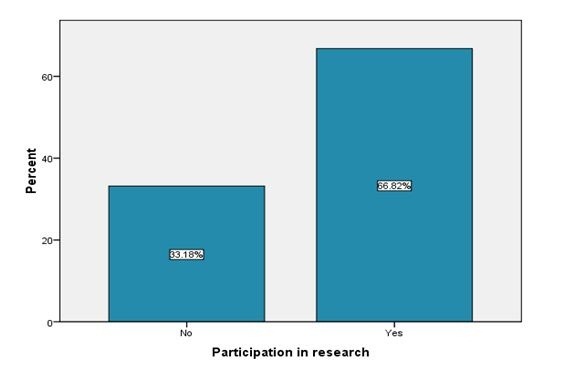
Distribution of respondents by participation in research for schistosomiasis and soil transmitted helminths

Assessment of household survey showed that 178 (82%) of the respondents received treatment with albendazole and praziquantel for intestinal worms and urogenital schistosomiasis while 39(18%) did not receive treatment (Table 2). There was a significant association between having received treatment and participation in the research (χ^2^= 27.811, df = 1, P > 0.05). Majority of those who received treatment133 (74.7%) participated in the research while 45 (25.2%) did not participate in the research. 26 (66.7%) respondents who never received treatment did not participate in the research while only 13 (33.3%) of those who never received treatment participated in the research. This was confirmed during qualitative data analysis from in-depth interviews. One of the key informants reported; “*I usually contribute samples for research because I received the treatment and I have seen those Kemri people here severally so I think it is important*”. This could be the one of the reasons why many of the participants who had participated in the treatment programme also participated in research. Most volunteers (Community health workers and village elders) who distributed drugs were less confident because they were not from this area and they did not possess enough information on control of urogenital schistosomiasis and intestinal worms in relation to the programme. One of the CHW during an indepth interview stated that *"there are still gaps on community acceptance of such programme as the community is not fully reached and the CHWs and village elders are also challenged by the community especially during the mobilization of the community to give samples for research"*. This is because they have not been trained well on issues of concern on urogenital schistosomiasis and STH. Majority of the respondents 146 (67%) participated in the contribution of samples required for the research while only compared to (32.7%) who did not contribute (Figure 2). There was a significant relationship between awareness of research and respondents participation in research (χ^2^= 1.115, df = 1, p = 0.001) with majority (85.2%) of those who were aware of the research having participated. Those who participated in the research gave (urine, blood and stool) which were necessary for the investigations. The respondents indicated that the treatment programme was of great help to them because since it started the morbidity especially in school children has greatly reduced as evidenced by their attendance in school compared to earlier times when most of the children missed school classes.

**Table 2:** Distribution of respondents who received treatment for urogenital schistosomiais et hookworm infections

Treatment of urogenital schistosomiasis and intestinal worms	N = 217	%	P value
**Respondent received treatment in January 2012**			
Yes	178	82	P= 0.00
No	39	18	
**Those who administered the drugs**			
Community health worker	124	69.7	
Doctor	4	2.2	
Teacher	50	28.1	
**Reason for not receiving treatment**			
It was for school children	4	10.3	
Thought they were family planning drugs	1	2.6	
Was never given	17	43.6	
Was not involved	14	35.9	
Was pregnant	3	7.7	

## Discussion

Community characteristics (demography, socio-economic, health seeking behaviour and priorities) may affect the role any community might play in a disease control programme. There is need for an assessment to be conducted before a control programme is implemented as this may vary across the communities. In order to realize full community participation in a programme continued interaction and communication with the beneficiaries is critical. The current study indicates that community's socio-economic situation and understanding of the disease has an influence in the search for treatment and participation in research. Socioeconomic status revealed to have an influence on treatment seeking behaviour and participation in the research as those who had low income, less education and no occupation were less likely to have good knowledge of urogenital schistosomiasis and intestinal worms, less likely to seek treatment, less likely to have a latrine or use it and less likely to participate in the research. Therefore, the need of education strategies aiming at providing skills and resources such as helping in construction of latrines and health risks attributed to these infections. A previous study conducted in Ghana to assess health seeking behaviour and utilization of health for schistosomiasis related symptoms revealed that people with higher socio-economic status sought health care more frequently than people with low socio-economic status [12]. Gender, age and family size did not have a significant association with the community's participation in the research and treatment programme though there are existing studies which have shown influence of gender in participation of treatment programmes of infectious diseases. In a study of community perceptions of intestinal schistosomiasis it was noted that the disadvantaged socio-economic status of women in rural communities of Uganda prevented them from participating in health programmes and accessing information on control or preventive measures for the disease [13].

The current study shows that the community heavily relies on mass drug administration. To overcome this situation there is need of increasing community based directed services on control, prevention and treatment of schistosomiasis and intestinal worms through engagement of the community directly. Health seeking behaviour is partly linked to people's beliefs and practices such that most of them end up seeking treatment from the herbalists (use of pawpaw roots concoction) as reported by one of the village elders. The findings concur with a study conducted in Tanzania where different ethnic groups tend to favor treatment at home by using various medicinal plants to treat cases of haematuria (*S. haematobium*) and thus do not result to medical treatment [14]. The community has prioritized witchcraft in that they associate the symptoms of these infections with witchcraft e.g. painful urination and when the child has intestinal worms they seek treatment from the witchdoctors in search of a reverse of the child's condition. The long distances, lack of money and time widely contributes to their behavior of failing to seek treatment in the face of disease thus the free treatment under the programme acts as a motivational factor.

The treatment coverage with albendazole and praziquantel was high with coverage rate of 82%; this might have a significant impact on the level of intensities of urogenital schistosomiasis and hookworms among the adult population of this community. This might provide a cost-effective treatment strategy for similar national control programmes in resource poor settings and endemic areas. A similar strategy was successfully used to control urogenital schistosomiasis in Burkina Faso [15]. The high treatment coverage of urogenital schistosomiasis and hookworms was likely due to opportunities by those who distributed the drugs to make frequent home visits in the households. The study gives similar findings on treatment coverage of a study by Ndyomugyenyi and Kabatereine [16] which showed that community directed interventions achieved higher treatment coverage (85%) for praziquantel and albendazole when the other risk groups in the community was involved. However, there are a number of factors which were identified in the study as a hindrance to acceptance of the treatment by the community members which should be addressed by stakeholders planning to undertake similar programmes in other endemic areas in order to realize full coverage. The house-to-house distribution strategy was noted to be placing a burden as most community members were often unaware of the treatment programme and could not plan to stay at home and wait to be treated. Another important observation was that the distances between households which are far, rumours that the treatment with praziquantel and albendazole being given to the community were for family planning. Health communication among the community members is the key in underpinning such perceptions, raising awareness of the health risks and importance of treatment.

Community based treatment may also be less successful than expected in case of low commitment by community leaders and low priority given to helminthes control by communities, or if there is the perception that intervention programmes should be the responsibility of the regulated health services rather than the responsibility of the community [17]. This study reveals that the community has a huge responsibility for such intervention programmes to be fully successful and there is need to prioritize on the local community members and leaders involvement of the so that they can be part of the programme. One of the key outcomes of this study is that control programmes should be accompanied with health education important for change in behavior, clear identification of the target population and take local knowledge and perceptions into consideration [18]. In this context consultation is important in disease control for programmes to be effective therefore the need to involve the community from design and intiation of the programme. This study also reveals the local understanding of the community on socio-economic and cultural factors which are important in designing programmes. A previous study suggested that projects must be built on an understanding of social, cultural, economic and political factors and upon lay knowledge and perceptions for effectiveness [19]. Anticipating community participation in a programme is not an intelligent guess, as this is a learning process for beneficiaries and the stakeholders which can be earned through the sharing of experiences by all the concerned actors.

## Conclusion

In conclusion there is need for continuous advocacy and dissemination of information to the communities to enhance community awareness and participation in similar control programmes. Further research needs to be conducted, at national level and other communities especially in other endemic areas in order to get a clear picture of people's behavior, knowledge, perception and other factors which may influence similar programmes targeted to include the adult population.

### What is known about this topic

- There are improvements which have been made to reduce helminthic transmission but worms’ infections continue to be an issue of major public health and socio-economic concern;
- There is still lack of good understanding of community response and behaviour when presented with a disease control strategy with which community members are expected to comply. A study conducted in Zimbabwe revealed that community participation is a complex process upon which a multiplicity of social and cultural determinants has an impact;
- Community participation in a disease control programme has been viewed as a mutual learning process where obstacles are identified and discussed and solutions shared among community members and staff (20).

### What this study adds

- The study clarifies factors which may hinder implementation of effective community based programmes which may help practitioners to implement meaningful interventions;
- The findings of this study also provide insight on areas that need to be addressed by programme implementers in the planned scaling up of control programs for schistosomiasis and STH in many endemic areas;
- The study findings are also useful in articulating guidelines on control and elimination programs for schistosomiasis and STH.
